# Accessing Colony Boundary Strengthening of Fully Lamellar TiAl Alloys via Micromechanical Modeling

**DOI:** 10.3390/ma10080896

**Published:** 2017-08-03

**Authors:** Jan Eike Schnabel, Swantje Bargmann

**Affiliations:** 1 Institute of Materials Research, Materials Mechanics, Helmholtz-Zentrum Geesthacht, Max-Planck-str. 1, 21502 Geesthacht, Germany; 2 Chair of Solid Mechanics, University of Wuppertal, Gaussstr. 20, 42119 Wuppertal, Germany; bargmann@uni-wuppertal.de

**Keywords:** TiAl, fully lamellar, crystal plasticity, Hall–Petch, polysynthetically twinned crystal

## Abstract

In this article, we present a strategy to decouple the relative influences of colony, domain and lamella boundary strengthening in fully lamellar titanium aluminide alloys, using a physics-based crystal plasticity modeling strategy. While lamella and domain boundary strengthening can be isolated in experiments using polysynthetically twinned crystals or mircomechanical testing, colony boundary strengthening can only be investigated in specimens in which all three strengthening mechanisms act simultaneously. Thus, isolating the colony boundary strengthening Hall–Petch coefficient $K_{C}$ experimentally requires a sufficient number of specimens with different colony sizes $\lambda_{C}$ but constant lamella thickness $\lambda_{L}$ and domain size $\lambda_{D}$, difficult to produce even with sophisticated alloying techniques. The here presented crystal plasticity model enables identification of the colony boundary strengthening coefficient $K_{C}$ as a function of lamella thickness $\lambda_{L}$. The constitutive description is based on the model of a polysynthetically twinned crystal which is adopted to a representative volume element of a fully lamellar microstructure. In order to capture the micro yield and subsequent micro hardening in weakly oriented colonies prior to macroscopic yield, the hardening relations of the adopted model are revised and calibrated against experiments with polysynthetically twinned crystals for plastic strains up to 15%.

## 1. Introduction

After decades of academic and industrial research, γ-based fully lamellar titanium aluminides (TiAl) outperform most competing high-temperature lightweight materials up to temperatures around 800 ${}^{\circ }$C [1,2,3]. Their exceptional properties originate from the dense arrangement of three types of microstructural boundaries, namely lamella, domain and colony boundaries [4,5]. Despite advances in understanding the three corresponding Hall–Petch effects, their relative contributions to the strength of fully lamellar TiAl is not yet consistently quantified. Micromechanical modeling helps to separate and thus quantify these effects as shown in the following.

### 1.1. Microstructure and Micromechanics of Fully Lamellar TiAl

#### 1.1.1. Lattice Structures and Orientation Relation

**Figure 1:**
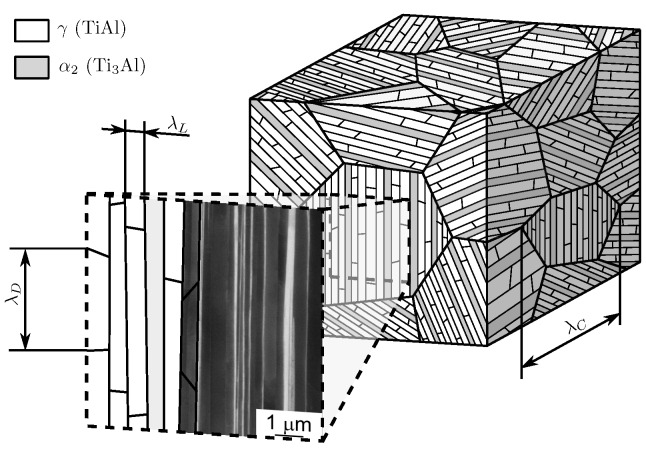
Schematic illustration of fully lamellar microstructure with magnification of lamellae. $\lambda_{L}$: lamella thickness; $\lambda_{D}$: domain size; $\lambda_{C}$: colony size. (SEM micrograph: courtesy of Michael Oehring, Helmholtz-Zentrum Geesthacht)

**Figure 2:**
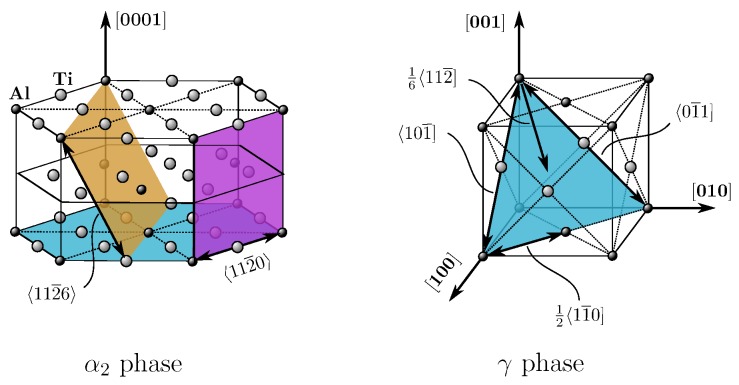
Crystallographic lattices of constituent phases with most relevant deformation mechanisms. left: hexagonal D0${}_{19}$ lattice of $\alpha_{2}$ phase (Ti${}_{3}$Al); right: tetragonal L1${}_{0}$ lattice of γ phase (TiAl).

Figure 1 schematically illustrates a fully lamellar microstructure of γ-based TiAl with its grain-shaped colonies, each subdivided into countless γ lamellae with a minor fraction of dispersed $\alpha_{2}$ lamellae. The crystallographic lattices of both phases are depicted in Figure 2.

The tetragonal L1${}_{0}$ lattice of the γ phase exhibits four twinning systems $\frac{1}{6}\langle 11\bar{2}]$, four ordinary slip systems $\frac{1}{2}\langle 1\bar{1}0]$ and eight super slip systems, namely all systems involving a *c* component. Plastic deformation of the hexagonal D0${}_{19}$ lattice of the $\alpha_{2}$ phase is carried by prismatic, pyramidal and basal slip. Table 1 lists respective deformation mechanisms for both phases.

The lattices of the $\alpha_{2}$ and γ lamellae in each colony are aligned following the orientation relation ${\left(111\right)}_{\gamma }∥{\left(0001\right)}_{\alpha_{2}}$ allowing coexistence of six orientation variants of the γ lattice that manifest in the domain structure, shown in Figure 1 (see, e.g., [7] for details). Separation distances of lamella, domain and colony boundaries are denoted by $\lambda_{L}$, $\lambda_{D}$ and $\lambda_{C}$ throughout this paper.

#### 1.1.2. Influence of Microstructural Boundaries on Strength

There is general agreement that colony, lamella and domain boundaries all give rise to Hall–Petch strengthening [4,5,8,9,10,11,12,13,14,15,16,17]. Thus, the yield strength of fully lamellar TiAl is a function of three Hall–Petch slopes *K* and the inverse square roots of corresponding microstructural lengths:

(1) $$\sigma_{Y}=f\left(\frac{K_{C}}{\sqrt{\lambda_{C}}},\frac{K_{D}}{\sqrt{\lambda_{D}}},\frac{K_{L}}{\sqrt{\lambda_{L}}}\right).$$

**Figure 3:**
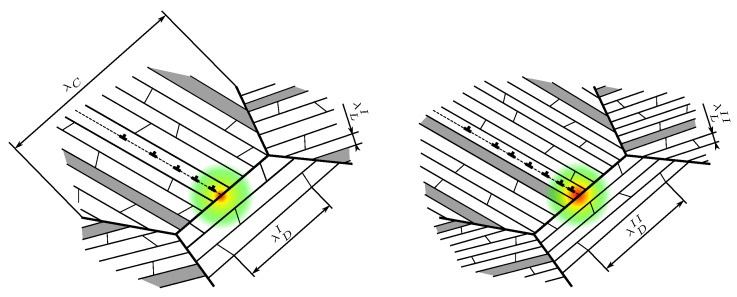
Illustration of dislocation pile-up stress at a colony boundary possibly activating slip/twinning in the adjacent colony. For $\lambda_{C}^{II}=\lambda_{C}^{I}$ but $\lambda_{L}^{II}<\lambda_{L}^{I}$ and $\lambda_{D}^{II}<\lambda_{D}^{I}$, the strength of respective systems in the right image will be higher than in the left one, requiring a higher pile-up stress to be activated and thus making colony boundary strengthening a function of $\lambda_{L}$ and $\lambda_{D}$.

The Hall–Petch slopes $K_{L}$ and $K_{D}$ for lamella and domain boundary strengthening can be separately determined from experiments with polysynthetically twinned crystals (i.e., specimens that only contain parallel lamellae of one specific orientation) [8,11,18,19] or by micromechanical testing [20,21]. Colony boundary strengthening, however, can exclusively be investigated in specimens in which naturally all three strengthening mechanisms act simultaneously. Isolating the corresponding Hall–Petch slope $K_{C}$ thus requires a sufficient number of specimens with different colony sizes but (at least nearly) the same lamella thickness and domain size, hard to produce even with sophisticated alloying techniques [13,22]. Thus, only few of the reported $K_{C}$ values [10,13,14,16,17,23,24] were determined meeting this demand. In fact, the domain size $\lambda_{D}$ was given in neither of the cited references, but, since it is suspected to be correlated to lamella thickness $\lambda_{L}$ [12,25], specimens with the same $\lambda_{L}$ should exhibit similar values of $\lambda_{D}$. However, the $K_{C}$ values determined for (nearly) constant $\lambda_{L}$ [13,14,16]—and thus constant $\lambda_{D}$—still differ significantly for different values of $\lambda_{L}$ and $\lambda_{D}$. Understanding colony boundary strengthening as the dislocation pile-up stress, required to activate slip/twinning in an adjacent colony (cf. Figure 3), supports the idea of a functional relation between colony boundary strength and strength of respective slip/twinning systems as suggested in [12].

Consequently, this implies that

- $K_{C}$ is a function of $\lambda_{L}$ and $\lambda_{D}$, since strengths of slip/twinning systems in adjacent colonies are determined by lamella and domain boundary strengthening and
- experimentally determined $K_{C}$ values are only valid for the given combination of $\lambda_{L}$ and $\lambda_{D}$, rendering identification of the functional relation $K_{C}=f\left(\lambda_{L},\lambda_{D}\right)$ unreasonably labor-intensive.

With these implications, reported experimental values for the Hall–Petch slope $K_{C}$ may be regarded as a few points in a space spanned by $K_{C}$, $\lambda_{L}$ and $\lambda_{D}$, insufficient to reveal the functional relation $K_{C}=f\left(\lambda_{L},\lambda_{D}\right)$.

### 1.2. Scope of Present Paper

Other than in experiments, the choice of any specific combination of colony size, domain size and lamella thickness is not restricted in numerical simulations. Therefore, a well designed micromechanical model can be used to reveal $K_{C}=f\left(\lambda_{L},\lambda_{D}\right)$, finally enabling microstructure sensitive prediction of macroscopic yield stress of fully lamellar TiAl alloys.

While capturing selected micromechanical effects like, e.g., the plastic anisotropy of polysynthetically twinned crystals, the micromechanical models set up in the past (e.g., [13,26,27,28,29,30,31,32,33,34,35,36,37,38,39]) are limited to isothermal conditions and/or temperatures near room temperature. Furthermore, the model parameters in these formulations are generally found for one specific combination of temperature and microstructural lengths, necessitating recalibration of the model whenever the mechanical behavior shall be analyzed at different temperatures or for a different set of microstructural parameters. Therefore, we set up a thermomechanically coupled crystal plasticity model of a polysynthetically twinned crystal that incorporates lamella and domain boundary strenghtening as well as the typical yield stress temperature anomaly to enable yield stress prediction between room temperature and 900 ${}^{\circ }$C [18].

In the present paper, we aim to adapt this model to a representative volume element (RVE) of a polycolony fully lamellar microstructure in order to identify the functional relation $K_{C}=f\left(\lambda_{L},\lambda_{D}\right)$. Since a pronounced microyield has been observed in weakly oriented colonies prior to macroscopic yield (cf. digital image correlation (DIC) analyses in [40]), the hardening relations of the constitutive model [18] are revised to capture hardening of single colonies/polysynthetically twinned crystals up to several percent of plastic strain. The revised hardening model is subsequently calibrated against the uniaxial compression experiments with polysynthetically twinned crystals reported in [9].

## 2. Modeling

In this section, the thermomechanically coupled crystal plasticity model from [18] is revisited and extended in order to capture above mentioned microhardening as well as colony boundary strengthening.

### 2.1. Kinematics and Stress Measures

With the multiplicative split of the deformation gradient F into an elastic part $F_{E}$ and a plastic part $F_{P}$

(2) $$F=F_{E}\cdot F_{P},$$

the elastic representation of the right Cauchy–Green tensor $C_{E}$ can be defined as strain measure via

(3) $$\begin{array}{cc} C_{E} & =F_{E}^{T}\cdot F_{E} \end{array}$$

and the second Piola–Kirchhoff stress S reads

(4) $$S=J_{E}F_{E}^{-1}\cdot \sigma \cdot F_{E}^{-T}.$$

Here, $J_{E}=\det F_{E}$ is the Jacobian and σ the Cauchy stress. Correspondingly, the Mandel stress reads

(5) $$M=C_{E}\cdot S.$$

### 2.2. Thermomechanics and Temperature Evolution

The procedure of thermomechanical coupling follows the line of arguments in, e.g., [41,42,43] and is thus just briefly recalled here. The Helmholtz free energy ψ is assumed to be a function of $C_{E}$, absolute temperature θ and plastic internal variables $q_{n}$. It is additively split into an elastic and a plastic part, reading

(6) $$\psi \left(C_{E},\theta ,q_{n}\right)=\psi_{E}\left(C_{E},\theta \right)+\psi_{P}\left(\theta ,q_{n}\right).$$

As derived in, e.g., [41], introducing the relation between Helmholtz free energy ψ, internal energy ε and entropy η in the form ψ=ε−ηθ and inserting the corresponding time derivative $\dot{\psi }=\dot{\varepsilon }-\dot{\eta }\theta -\eta \dot{\theta }$ and subsequently the balance of internal energy into the Clausius–Duhem inequality, yields the following relations for stress and entropy:

(7) $$\begin{array}{cc} S & =2\rho_{0}\frac{\partial \psi }{\partial C_{E}}, \end{array}$$

(8) $$\begin{array}{cc} \eta & =-\frac{\partial \psi }{\partial \theta }, \end{array}$$

with $\rho_{0}$ being the density in reference configuration. Furthermore, the dissipation *D* reads

(9) $$D=\underset{D_{\mathrm{mech}}}{\underset{︸}{M:L_{P}-\rho_{0}\sum_{n}\frac{\partial \psi }{\partial q_{n}}{\dot{q}}_{n}}}-\underset{D_{\mathrm{therm}}}{\underset{︸}{\frac{Q}{\theta }\cdot \nabla_{0}\theta }}\geq 0.$$

In this, $L_{P}$ denotes the plastic velocity gradient and Q is the heat flux vector described via Fourier’s law $Q=-\kappa \nabla_{0}\theta$. The first two terms in Equation (9) represent the mechanical dissipation $D_{\mathrm{mech}}$ during plastic deformation, while the last term denotes the thermal dissipation $D_{\mathrm{therm}}$ due to heat conduction.

To obtain the temperature evolution equation, the time derivative of the Helmholtz free energy is inserted into the energy balance together with Equations (7) and (8). With the specific heat capacity $c_{p}=-\theta \frac{\partial^{2}\psi }{\partial \theta^{2}},$ this yields

(10) $$\rho_{0}c_{p}\dot{\theta }=-\mathrm{Div}Q+\underset{D_{\mathrm{mech}}}{\underset{︸}{M:L_{P}-\rho_{0}\sum_{n}\frac{\partial \psi }{\partial q_{n}}{\dot{q}}_{n}}}+\rho_{0}r+\frac{1}{2}\theta \frac{\partial S}{\partial \theta }:{\dot{C}}_{E}+\rho_{0}\theta \sum_{n}\frac{\partial^{2}\psi }{\partial \theta \partial q_{n}}{\dot{q}}_{n},$$

with *r* being the external heat supply per unit mass. With evolving plasticity (${\dot{q}}_{n}>0$), $D_{\mathrm{mech}}$ is lowered, i.e., energy is stored in the plastic internal variables $q_{n}$ instead of being dissipated as heat. If the plastic internal variables decrease, e.g., due to thermally activated recovery (${\dot{q}}_{n}<0$), the before stored energy is released in the form of heat.

### 2.3. Crystal Plasticity

Crystal plasticity models are based on Schmid’s law, which defines the resolved shear stress $\tau_{\alpha }$ on slip system α via

(11) $$\tau_{\alpha }:=s_{\alpha }\cdot M\cdot n_{\alpha },$$

with $s_{\alpha }$ and $n_{\alpha }$ being slip direction and slip plane normal of the respective slip system.

Following the ideas from [44], mechanical twinning in the γ phase is treated as a unidirectional shear mechanism acting in the twinning plane and obeying Schmid’s law, i.e., Equation (11). This leads to the following definition of the plastic velocity gradient $L_{P}$ (cf. [44])

(12) $$\begin{array}{c} L_{P}={\dot{F}}_{P}\cdot F_{P}^{-1}=\left[1-f\right]\underset{\mathrm{slip}}{\underset{︸}{\sum_{\alpha }^{N^{\mathrm{sl}}}\nu_{\alpha }\left[s_{\alpha }⊗n_{\alpha }\right]}}+\underset{\mathrm{twinning}}{\underset{︸}{\sum_{\beta }^{N^{\mathrm{tw}}}\gamma_{T}g_{\beta }\left[s_{\beta }⊗n_{\beta }\right]}}. \end{array}$$

Herein, shear rate of slip system α and the twinning rate of twinning system β are denoted by $\nu_{\alpha }$ and $g_{\beta }$, the total twinned volume fraction is given by *f* and $\gamma_{T}$ denotes the material specific twinning shear. Other than in the original formulation of $L_{P}$ from [44], lattice reorientation and subsequent slip in twinned regions are neglected since twins in γ lamellae are generally very narrow [5].

#### 2.3.1. Flow and Twinning Rule

##### Slip

The flow rule relates the resolved shear stress $\tau_{\alpha }$ to the resulting shear rate $\nu_{\alpha }$ on slip system α via the viscoplastic powerlaw (cf. [45])

(13) $$\nu_{\alpha }=\nu_{0}{\left|\frac{\tau_{\alpha }}{\tau_{\alpha }^{Y}}\right|}^{n}\mathrm{sign}\left(\tau_{\alpha }\right).$$

In this, $\tau_{\alpha }^{Y}$ is the current critical resolved shear stress and $\nu_{0}$ and *n* are the reference shear rate and the strain rate sensitivity exponent.

##### Twinning

To ensure that twinning is unidirectional and that the twinned volume fraction does not exceed the theoretical limit of f=1.0, the relation of resolved shear stress $\tau_{\beta }$ and twinning rate $g_{\beta }$ is modeled via (cf. [44])

(14) $$g_{\beta }=\left\{\begin{array}{cc} \frac{\nu_{0}}{\gamma_{T}}{\left[\frac{\tau_{\beta }}{\tau_{\beta }^{T}}\right]}^{n} & \mathrm{for}\;\tau_{\beta }>0\;\mathrm{and}\;f<1.0,\\ 0, & \mathrm{else}. \end{array}\right.$$

Herein $\tau_{\beta }^{T}$ denotes the current twinning resistance of twinning system β and the reference twinning rate is determined by dividing the reference shear rate $\nu_{0}$ by twinning shear $\gamma_{T}$ [44,46].

#### 2.3.2. Defect Density Evolution

In crystal plasticity, plastic deformation is represented by the accumulated shear of underlying deformation mechanisms instead of discretely resolving defects like dislocations or twins. The defect density evolution is, however, directly correlated to the stored energy of cold work, enables a consistent definition of dissipation and provides a physics-based way to describe work hardening and thermal recovery processes as, e.g., investigated in [15,47]. Therefore, we introduce the twinned volume fractions $f_{\beta }$ for twinning systems β and the dislocation densities $\rho_{\alpha }^{\mathrm{dis}}$ for slip systems α as well as the total dislocation density $\rho^{\mathrm{dis}}$ given by $\rho^{\mathrm{dis}}=\sum_{\alpha }^{N^{\mathrm{sl}}}\rho_{\alpha }^{\mathrm{dis}}$ and the total twinned volume fraction *f* (see Equation (12)), which is determined via $f=\sum_{\beta }^{N^{\mathrm{tw}}}f_{\beta }\leq 1.0$. Although not specifically intended here, a micromechanical model capable of tracking the evolution of these defect densities in TiAl alloys may, e.g., grant valuable insight into forming processes in which the defect density dictates necessary forming forces and the number of annealing steps [47].

##### Dislocation Density Evolution

The dislocation densities $\rho_{\alpha }^{\mathrm{dis}}$ are assumed to evolve with the generation/recovery formulation (cf. [48,49,50])

(15) $${\dot{\rho }}_{\alpha }^{\mathrm{dis}}=A_{\alpha }\left(\rho_{\alpha }^{\mathrm{dis}}\right)\left|\nu_{\alpha }\right|-R_{\alpha }\left(\rho_{\alpha }^{\mathrm{dis}},\theta \right).$$

The first term on the right side represents the dislocation generation due to shear on slip system α ($|\nu_{\alpha }|>0$). Herein, $A_{\alpha }$ is described by the saturation relation [48,49]

(16) $$A_{\alpha }=A_{\alpha ,0}\left[1-{\left[\frac{\rho_{\alpha }^{\mathrm{dis}}}{\rho_{\alpha ,\mathrm{sat}}^{\mathrm{dis}}}\right]}^{p_{\alpha }}\right],$$

where $A_{\alpha ,0}$ is the reference accumulation coefficient, $\rho_{\alpha ,\mathrm{sat}}^{\mathrm{dis}}$ is the saturation value for dislocation density and $p_{\alpha }>0$ is a constant.

The second term in Equation (15) describes static thermal recovery and follows an Arrhenius type law (cf. [47,49])

(17) $$R_{\alpha }=R_{\alpha ,0}\exp\left(-\frac{Q_{R}}{k_{B}\theta }\right){\langle \frac{\rho_{\alpha }^{\mathrm{dis}}-\rho_{\alpha ,\min}^{\mathrm{dis}}}{\rho_{\mathrm{ref}}^{\mathrm{dis}}}\rangle }^{q_{\alpha }},$$

where $R_{\alpha ,0}$ denotes the reference recovery rate, $Q_{R}$ is the activation energy for recovery, $k_{B}$ denotes the Boltzmann constant, $\rho_{\alpha ,\min}^{\mathrm{dis}}$ is the minimum dislocation density for recovery to take place and $\rho_{\mathrm{ref}}^{\mathrm{dis}}$ is a reference dislocation density. The exponent $q_{\alpha }>0$ is a constant.

##### Twin Evolution

In the context of thermomechanical modeling, it has to be noted, that—other than in conventional metallic materials—twinning is not only a room temperature mechanism in TiAl alloys. In fact, twinning is rather pronounced at elevated temperatures and even plays a significant role in creep deformation [5,51]. Therefore, twinning is not restricted to room temperature in this model. The twinned volume fractions $f_{\beta }$ are assumed to evolve directly with the corresponding twinning rates, i.e.,

(18) $${\dot{f}}_{\beta }=g_{\beta }.$$

#### 2.3.3. Critical Resolved Shear Stresses

The slip and twinning system strengths $\tau_{\alpha }^{Y}$ and $\tau_{\beta }^{T}$ from Equations (13) and (14) can be written as follows:

(19) $$\begin{array}{c} \tau_{\alpha }^{Y}=\tau_{\alpha ,0}^{Y}+\Delta \tau_{\alpha }^{Y}, \end{array}$$

(20) $$\begin{array}{c} \tau_{\beta }^{T}=\tau_{\beta ,0}^{T}+\Delta \tau_{\beta }^{T}. \end{array}$$

Here, $\tau_{\alpha ,0}^{Y}$ and $\tau_{\beta ,0}^{T}$ are the temperature and micro structure dependent initial slip and twinning system strengths and $\Delta \tau_{\alpha }^{Y}$ and $\Delta \tau_{\beta }^{T}$ denote their evolution due to plastic deformation, i.e., represent work hardening.

##### Initial Slip/Twinning System Strength

As mentioned earlier, the yield strength of fully lamellar TiAl alloys is mainly determined by three coexisting Hall–Petch effects. Depending on its orientation with respect to the lamellae, a slip/twinning systems shear plane does either cross the lamella or the domain boundaries. Respectively, either $\lambda_{L}$ or $\lambda_{D}$ is the determining microstructural length for Hall–Petch strengthening [6,8,19]. The colony size $\lambda_{C}$, however, has the same influence on all slip/twinning systems independent of their orientation. The initial slip/twinning system strengths can thus be written as

(21) $$\begin{array}{c} \tau_{\alpha ,0}^{Y}=\tau_{R}+\frac{k_{D/L}}{\sqrt{\lambda_{D/L}}}+\frac{k_{C}}{\sqrt{\lambda_{C}}}, \end{array}$$

(22) $$\begin{array}{c} \tau_{\beta ,0}^{T}=\tau_{R}+\frac{k_{D/L}}{\sqrt{\lambda_{D/L}}}+\frac{k_{C}}{\sqrt{\lambda_{C}}}, \end{array}$$

with $\tau_{R}$ being the lattice resistance to slip/twinning. The here introduced Hall–Petch coefficients *k* are defined on the slip/twinning system level and are not directly comparable with the measured Hall–Petch coefficients *K*. In polysynthetically twinned crystals, however, $k_{D}$ and $k_{L}$ may be determined from measured $K_{D}$ and $K_{L}$ values using the Schmid factors of selectively activated slip/twinning systems in certain orientations of the lamella plane with respect to load (cf. e.g., [18]). The last terms in Equations (21) and (22) are obviously only meaningful when modeling polycolony microstructures and thus have to be set to zero for simulations of polysynthetically twinned crystals.

The temperature dependence of $\tau_{\alpha ,0}^{Y}$ and $\tau_{\beta ,0}^{T}$ of the γ phase—especially the typical yield stress temperature anomaly—is incorporated in the parameters in Equations (21) and (22) as shown in [18]. The temperature dependent initial critical resolved shear stresses of the $\alpha_{2}$ phase are modeled according to [18], thus not involving the Hall–Petch strengthening shown in Equation (21).

##### Evolution of Slip System Strength

Once the resolved shear stress $\tau_{\alpha }$ exceeds $\tau_{\alpha ,0}^{Y}$, the slip systems strength increases due to dislocation interaction and nucleation of twins. In the reported crystal plasticity models of fully lamellar TiAl, work hardening was usually treated in a simplified way using, e.g., linear [26,29,31,32,33,35,37] or hyper secans [36,39,46] hardening laws, mostly neglecting the interaction with and between evolving twins. This proved sufficient for modeling the yield point of polysynthetically twinned crystals since, in this case, the plastic strains remain small. The micro yield in weakly oriented colonies, however, involves considerable local plastic strains. With the dislocation densities and twinned volume fractions, introduced earlier, we may thus set up a more physical, defect density-based work hardening description.

In general, hardening $\Delta \tau_{\alpha }^{Y}$ of slip system α can be expressed by

(23) $$\Delta \tau_{\alpha }^{Y}=\Delta \tau_{\alpha ,s|s}^{Y}+\Delta \tau_{\alpha ,s|t}^{Y},$$

where $\Delta \tau_{\alpha ,s|s}^{Y}$ denotes strengthening due to dislocation interactions and $\Delta \tau_{\alpha ,s|t}^{Y}$ denotes strengthening of slip system α by twin activity.

The slip|slip interaction $\Delta \tau_{\alpha ,s|s}^{Y}$ is best described in terms of the dislocation density via the well-known relation

(24) $$\Delta \tau_{\alpha ,s|s}^{Y}=aGb_{\alpha }\sqrt{\rho^{\mathrm{dis}}}.$$

Herein, $G=\frac{E}{2[1+\nu ]}$ denotes the shear modulus determined in terms of Young’s modulus *E* and Poisson’s ratio ν and $b_{\alpha }$ is the length of the Burgers vector of system α. The constant coefficient a≈0.5 in TiAl alloys [5].

The boundaries of evolving twins β act as strong barriers to dislocation motion (cf. e.g., [52,53]), thus reducing the free path length of non-coplanar (ncp) slip systems α (i.e., $n_{\alpha }∦n_{\beta }$). Since the representation of twins by their volume fraction neither provides information about their number nor their thickness, the resultant strengthening of non-coplanar slip systems cannot be modeled as function of the inverse square root of the actual free path length as done in e.g. [54,55]. Thus, several authors introduced formulations to account for this source of Hall–Petch type strengthening as function of twinned volume fraction [27,52,56]. Based on the formulation from [27], we introduce the following relation for the corresponding term $\Delta \tau_{\alpha ,s|t}^{Y}$

(25) $$\Delta \tau_{\alpha ,s|t}^{Y}=\frac{\sum_{\beta }^{\mathrm{ncp}}h_{\alpha \beta }f_{\beta }}{1.0-\sum_{\beta }^{\mathrm{ncp}}f_{\beta }},$$

with $h_{\alpha \beta }$ being a coefficient for hardening of slip system α due to twinning on non-coplanar system β.

##### Evolution of Twinning System Strength

The strength $\tau_{\beta }^{T}$ of twinning system β increases with nucleation of non-coplanar twins (i.e., $n_{\beta }∦n_{\beta^{\prime }}$) and with interaction of twinning dislocations with the slip dislocation network [55]. In a general form, this can be written as

(26) $$\Delta \tau_{\beta }^{T}=\Delta \tau_{\beta ,t|t}^{T}+\Delta \tau_{\beta ,t|s}^{T},$$

introducing $\Delta \tau_{\beta ,t|t}^{T}$ as the strengthening due to nucleation of non-coplanar twins and $\Delta \tau_{\beta ,t|s}^{T}$ to account for interaction of twinning dislocations with slip dislocations.

Hall–Petch strengthening of twinning system β by non-coplanar twins $\beta^{\prime }$ is modeled as for slip systems, reading

(27) $$\Delta \tau_{\beta ,t|t}^{T}=\frac{\sum_{\beta^{\prime }}^{\mathrm{ncp}}h_{\beta \beta^{\prime }}f_{\beta^{\prime }}}{1.0-\sum_{\beta^{\prime }}^{\mathrm{ncp}}f_{\beta^{\prime }}}.$$

Herein, $h_{\beta \beta^{\prime }}$ is again a hardening coefficient.

As described in [55], twinning dislocations may interact with slip dislocations, although this effect is not as severe as the other strengthening mechanisms. The corresponding formulation from [55] reads

(28) $$\Delta \tau_{\beta ,t|s}^{T}=Gb_{\beta }\sum_{\alpha }^{N^{\mathrm{sl}}}C_{\beta \alpha }b_{\alpha }\rho_{\alpha }^{\mathrm{dis}},$$

where $C_{\beta \alpha }$ accounts for interaction between twinning system β and slip system α.

**Figure 4:**
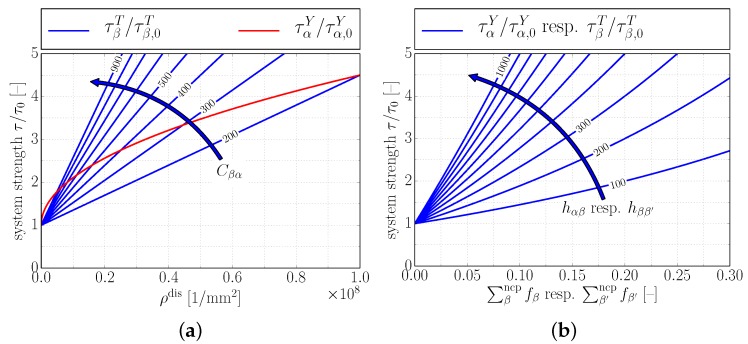
Qualitative illustration of hardening laws, i.e., Equations (24), (25), (27) and (28). (**a**) strengthening of slip systems α and twinning systems β with total dislocation density $\rho^{\mathrm{dis}}$; influence of interaction coefficient $C_{\beta \alpha }$; (**b**) strengthening of slip systems α and twinning systems β with volume fraction of non-coplanar twins; influence of hardening coefficient $h_{\alpha \beta }$ resp. $h_{\beta \beta^{\prime }}$.

Figure 4 qualitatively shows the evolution of slip/twinning system strength.

The hardening relations are not dependent on temperature, which is a reasonable assumption for the modeled defect structures, since they cannot be overcome by thermal activation [5].

### 2.4. Helmholtz Free Energy

As stated earlier, the Helmholtz free energy is assumed to be a function of the elastic Cauchy–Green strain tensor $C_{E}$, temperature θ and plastic internal variables $q_{n}$. The dislocation densities $\rho_{\alpha }^{\mathrm{dis}}$ are well suited plastic internal variables, since they are directly related to the stored energy of cold work, enabling a physically meaningful representation of mechanical dissipation and temperature evolution.

While the stored energy of cold work increases linearly with dislocation density, the energy stored in the form of twin/matrix interfaces is given by the fixed interface energy per twin boundary (i.e., the stacking fault energy) and, thus, does not change with twin growth [5]. Therefore, the discrete number of twins has to be known to correctly assess their contribution to stored energy of cold work. Since resolving the discrete number of twins is not possible with the here presented continuum theory, we neglect the corresponding contribution to stored energy of cold work by assuming that the Helmholtz energy is no function of the twinned volume fraction.

With these assumptions, the Helmholtz free energy from Equation (6) is rewritten as

(29) $$\rho_{0}\psi :=\rho_{0}\psi_{E}\left(C_{E},\theta \right)+\rho_{0}\psi_{P}\left(\rho_{\alpha }^{dis},\theta \right).$$

The elastic part of the Helmholtz free energy $\rho_{0}\psi_{E}$ is assumed to follow a Neo-Hookean behavior (cf. [18])

(30) $$\begin{array}{cc} \rho_{0}\psi_{E}= & \frac{\mu }{2}\left[\mathrm{tr}C_{E}-3\right]+\frac{\lambda }{2}\ln^{2}J_{E}-\mu \ln J_{E}-3\alpha_{t}K\left[\theta -\theta_{0}\right]\frac{\ln J_{E}}{J_{E}}\\ +\rho_{0}c_{p}\left[\theta -\theta_{0}-\theta \ln\frac{\theta }{\theta_{0}}\right]-\left[\theta -\theta_{0}\right]S_{0}, \end{array}$$

where $\mu =\frac{E}{2(1+\nu )}$ and $\lambda =\frac{\nu E}{\left(1+\nu )(1-2\nu \right)}$ are the Lamé constants, $\alpha_{t}$ denotes the thermal expansion coefficient, $K=\frac{E}{3-6\nu }$ is the bulk modulus and $S_{0}$ is the absolute entropy density. The plastic part of the Helmholtz free energy $\rho_{0}\psi_{P}$ reads [48,49]

(31) $$\rho_{0}\psi_{P}=aG\sum_{\alpha }^{N^{\mathrm{sl}}}b_{\alpha }^{2}\rho_{\alpha }^{\mathrm{dis}}.$$

With this definition of the Helmholtz free energy and relations (7) and (10), the stress and the temperature evolution read

(32) $$\begin{array}{cc} S= & \mu \left[I-C_{E}^{-1}\right]+\left[\lambda \ln J_{E}-\frac{3\alpha_{t}K}{J_{E}}\left[\theta -\theta_{0}\right]\left[1-\ln J_{E}\right]\right]C_{E}^{-1}, \end{array}$$

(33) $$\begin{array}{cc} \rho_{0}c_{p}\dot{\theta }= & -\mathrm{Div}Q+M:L_{P}-a\sum_{\alpha }^{N^{\mathrm{sl}}}\left[G-\theta \frac{dG}{d\theta }\right]b_{\alpha }^{2}{\dot{\rho }}_{\alpha }^{\mathrm{dis}}+\rho_{0}r+\frac{1}{2}\theta \frac{\partial S}{\partial \theta }:{\dot{C}}_{E}. \end{array}$$

### 2.5. RVE Generation and Discretization

In the following, two representative volume elements (RVEs) are set up to serve as geometries for the intended finite element analysis—one for a polysynthetically twinned crystal and one for a polycolony fully lamellar microstructure.

#### 2.5.1. RVE of a Polysynthetically Twinned Crystal

**Figure 5:**
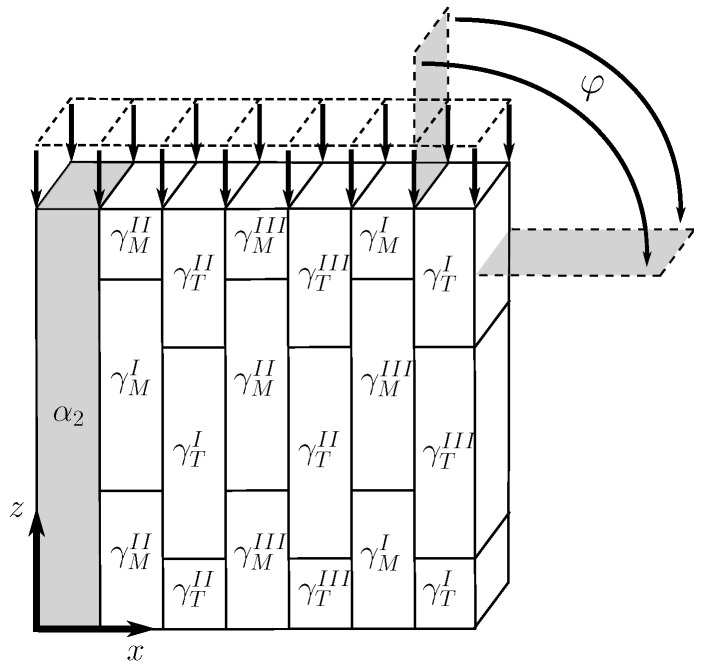
Representative volume element of polysynthetically twinned crystal. φ: angle between uniaxial load and lamella plane; $\gamma_{M/T}^{I-III}$: six orientation variants of γ phase (three matrix and three twin orientations).

As shown in [18,33], it is sufficient to discretize only seven lamellae to represent the geometry of a polysynthetically twinned crystal, i.e., one $\alpha_{2}$ lamella and one for each γ orientation variant. Figure 5 schematically depicts the chosen representative volume element.

This RVE is subjected to periodic boundary conditions and its rotation with respect to the uniaxial load is realized as described in [57]. The geometry is meshed using linear hexahedral elements.

#### 2.5.2. RVE of a Polycolony Microstructure

Due to the high aspect ratio of the lamellae and their large number per colony, a one-to-one discretization of a polycolony fully lamellar microstructure is computationally highly inefficient. Therefore, numerical homogenization schemes and/or geometrical simplifications are inevitable.

**Figure 6:**
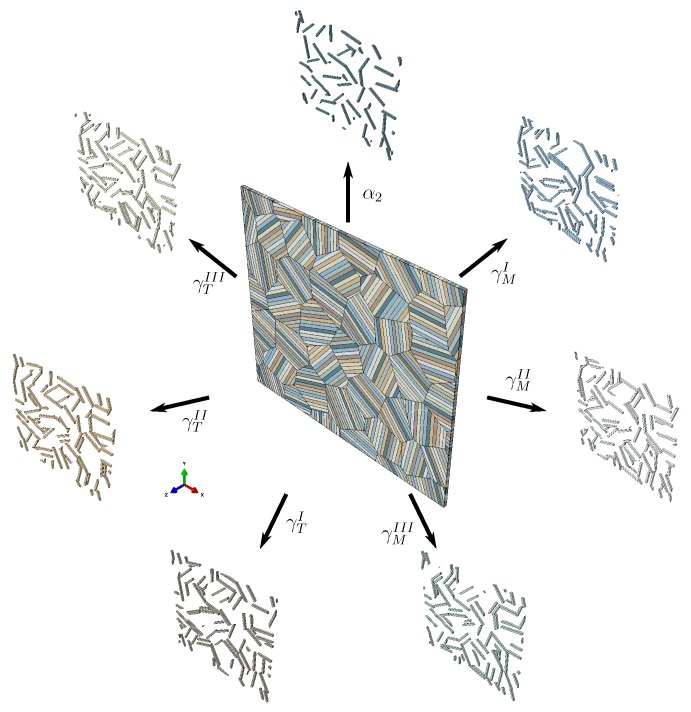
Representative volume element of polycolony fully lamellar microstructure consisting of 36 lamellar colonies. Separate depiction of the $\alpha_{2}$ phase and the orientation variants of the γ phase shows their distribution within the colonies.

We set up an RVE with a reduced number of lamellae per colony but ensure that the volume fractions of $\alpha_{2}$ and γ lamellae are correctly reflected in each colony. The RVE is shown in Figure 6.

The shapes of the 36 colonies in this RVE are based on a randomized 2D Voronoi diagram. Periodicity is achieved by repeating the diagram in a matrix pattern. The 2D Voronoi diagram is extruded in the *z*-direction, resulting in a columnar 3D representation of colonies. Subsequently, the lamella boundaries are introduced by Boolean intersection of appropriately oriented planes with the single columnar Voronoi cells. The orientation of the lamella plane in each colony is uniquely defined by only one angle $\varphi_{i}$ rotating around the *z*-axis. To minimize the influence of texture, the orientations of the colonies are evenly distributed between $\varphi_{i}=0^{\circ }$ and $\varphi_{i}=360^{\circ }$ (i.e., $\varphi_{i}=i\frac{360^{\circ }}{n_{\mathrm{col}}}$ for $i=1,2,...,n_{\mathrm{col}}$). Furthermore, the orientation of the γ phase along the lamellae is not altered, i.e., each lamella contains only one γ orientation.

This RVE is subjected to periodic boundary conditions and meshed using linear wedge elements.

## 3. Parameter Identification

The introduced defect density-based hardening model is best calibrated against experiments with polysynthetically twinned crystals or micropillar compression tests since both allow for investigating the micromechanical behavior of a single colony without the influence of neighboring colonies and colony boundaries. Although micropillar compression has the clear advantage of enabling the analysis of single colonies within the actual microstructure [20,21], currently more data is available on the plastic deformation of polysynthetically twinned crystals. In [9], the plastic deformation of polysynthetically twinned crystals is analyzed for eight different load angles between 0${}^{\circ }$ and 90${}^{\circ }$ and up to plastic strains of about 15% yielding a good data base for the here intended model calibration.

### 3.1. Constitutive Assumptions

#### 3.1.1. Morphological Classification

As stated in [6], all slip and twinning systems in fully lamellar TiAl can be uniquely classified according to their morphology to be either

- longitudinal (s∥ lamellar plane; n⊥ lamellar plane),
- mixed (s∥ lamellar plane; n 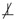 lamellar plane) or
- transversal (s∦ lamellar plane; n 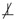 lamellar plane).

systems (cf. Table 1). As shown in [6] and later confirmed by other authors, e.g., [6,18,26,35,57], assigning the same model parameters to all slip and twinning systems of a morphological class effectively reproduces the plastic deformation behavior of the lamellar compound while leaving only three parameter sets that have to be identified instead of one per individual slip/twinning system.

#### 3.1.2. Hall–Petch Strengthening by Evolving Twins

**Figure 7:**
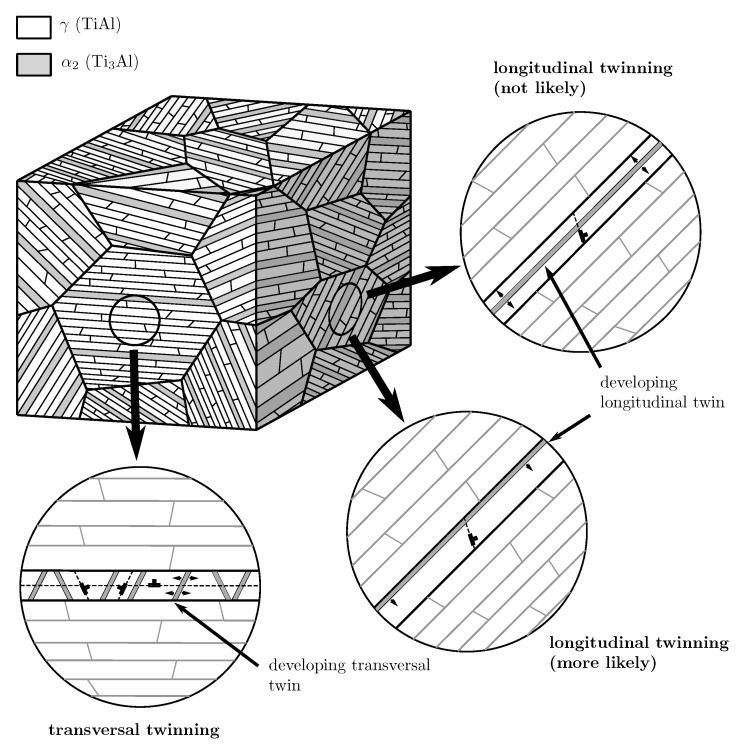
Comparison of possible evolutions of longitudinal and transversal twins in γ lamellae.

Due to their different orientations with respect to the lamella plane, evolving longitudinal and transversal twins in the γ lamellae presumably contribute to a different extent to the strength of non-coplanar slip and twinning systems. Transversal twins evolve as many thin needles that subdivide the γ lamellae [58] and, thus, strongly reduce the free path length of longitudinal and mixed deformation systems. Other transversal deformation systems do, however, not necessarily have to cross these twins (cf. Figure 7), consequently being strengthened by them to a lesser extent. Little is known about the evolution of longitudinal twins since they are harder to capture in experiments because they cannot be distinguished from original lamellae after plastic deformation. However, it is very unlikely that several longitudinal twins nucleate within a single lamella since this is energetically unfavorable. Longitudinal twins will more likely grow from an already existing lamella boundary slightly reducing the lamella thickness but most probably not causing excessive strengthening of non-coplanar deformation systems (cf. Figure 7). Therefore, we distinguish strengthening by longitudinal and transversal twinning in the here derived parameter set.

#### 3.1.3. Modeling Super Slip

##### Initial Critical Resolved Shear Stress

Since it has been frequently observed experimentally that super slip systems are less active than ordinary slip systems, they are suspected to exhibit a (slightly) higher critical resolved shear stress [4]. Unfortunately, the isolated critical resolved shear stress of super slip systems could not yet be accessed experimentally. Therefore, the contribution of super slip to the deformation of fully lamellar TiAl and appropriate modeling approaches have been discussed to some extent in the past. Some authors did, e.g., not allow any super slip in their models [59,60], introducing a pronounced tension/compression asymmetry [33] (due to the unidirectionality of twinning) that was not observed in experiment [61], while others scaled the strength of super slip systems by a factor $Q_{so}\geq 1$ with respect to the strength of ordinary slip systems [33,37]. Since there is no agreement on the actual strength of super slip systems, we choose a different approach here. To keep the benefit of Lebensohns morphological classification [6], the critical resolved shear stresses of all deformation mechanisms of a morphological class are assumed to be the same, neglecting the potentially higher stresses necessary to activate super slip.

##### Taylor Hardening

Lattice restoring super slip requires full $\langle 0\bar{1}1]$ translations and may, e.g., be achieved by two identical $\frac{1}{2}\langle 0\bar{1}1]$ super partial dislocations separated by an antiphase boundary [5]. Thus, Taylor hardening of super slip systems can be described using the Burgers vector of either the two individual super partial dislocations $\frac{1}{2}\langle 0\bar{1}1]$ or the perfect super dislocation $\langle 0\bar{1}1]$ in corresponding Equation (24). In order to account for the collective movement of the two super partial dislocations, bonded by the antiphase boundary, we use the Burgers vector of the perfect super dislocation. This results in a stronger hardening of super slip systems and, thus, reflects the fact that both super partial dislocations have to pass the forest of dislocations together. In consequence, ordinary slip is preferably activated in most crystallographic orientations as observed in experiments.

#### 3.1.4. Recovery

Since, for the moment, we are only interested in (temperature independent) work hardening behavior of polysynthetically twinned crystals, the recovery related terms in Equations (15) and (17) are neglected in the following.

### 3.2. Model Parameters

Some parameters of the presented crystal plasticity model can be determined directly from experiments or have well-established standard values from literature. The Hall–Petch coefficients $k_{D}$ and $k_{L}$ as well as the parameters for the viscoplatic flow rules (Equations (13) and (14)) are taken from our previous work [18]. Respective parameters are gathered in Table 2. The $\alpha_{2}$ content is not reported in [9], but, since the composition of the tested specimens is Al rich (Ti-49.3at %Al), we assume the $\alpha_{2}$ content to be as low as 2 Vol. % for the calibration process.

#### 3.2.1. Onset of Yield

**Figure 8:**
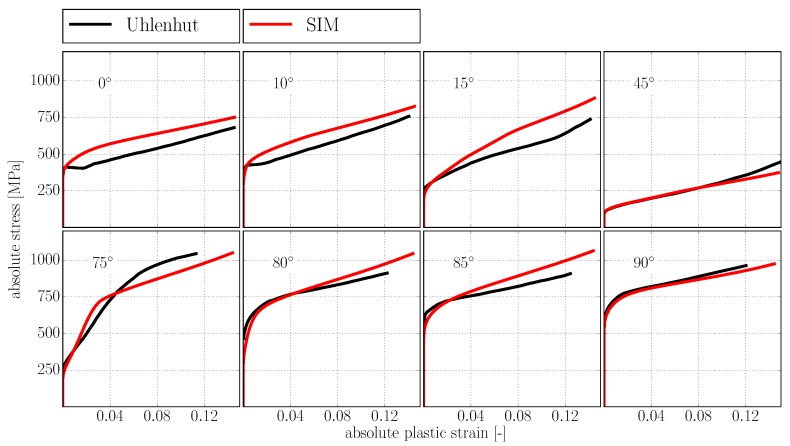
Orientation dependent yield and hardening behavior of polysynthetically twinned crystals. Experimental results taken from [9].

With the Hall–Petch coefficients from Table 2, solely lattice resistance $\tau_{R}$, domain size $\lambda_{D}$ and lamella thickness $\lambda_{L}$ remain to be identified for determining the initial critical resolved shear stresses $\tau_{\alpha ,0}^{Y}$ and $\tau_{\beta ,0}^{T}$ defined in Equations (21) and (22). Unfortunately, neither $\lambda_{D}$ nor $\lambda_{L}$ are reported in [9]. Furthermore, $\tau_{R}$ depends on multiple factors like e.g., composition and impurity of a given alloy and can thus not be identified universally. To calibrate the hardening model against the given data, we thus set $\tau_{R}=\;$25 MPa (based on experimental findings from [11]), $\lambda_{D}=\;$50 μm and $\lambda_{L}=\;$1 μm, which reproduces the initial yield of the experimental results from [9] reasonably well (see Figure 8).

#### 3.2.2. Dislocation Accumulation and Hardening Interaction

With above introduced constitutive assumptions and the fixed initial critical resolved shear stresses, the remaining parameters for dislocation accumulation (Equation (16)) and hardening (Equations (25), (27) and (28)) are found by successively adjusting them in trial simulations until the post yield behavior of the polysynthetically twinned crystals tested in [9] is reproduced well. Respective model parameters are gathered in Table 3.

### 3.3. Results

Comparison of the simulation results with experimentally determined stress strain curves from [9] is shown in Figure 8.

Given the high number of deformation systems involved in the plasticity of polysynthetically twinned crystals—namely six γ orientation variants with 12 slip systems and four twinning systems each as well as 12 slip systems for the $\alpha_{2}$ phase—the agreement between simulation and experimental results is very good. The qualitative features of the experimental curves are well reproduced, which is not possible with simple linear hardening relations as in [35,57]. We refrain from a quantitative improvement of the agreement between simulation and experiment by, e.g., numerical optimization methods, given the fact that in [9] only one specimen was tested per orientation.

## 4. Determining the Hall–Petch Coefficient for Colony Boundary Strengthening

With the calibrated defect density based model of a polysynthetically twinned crystal, the actual aim of the present paper is tackled: isolating the Hall–Petch coefficient for colony boundary strengthening $K_{C}$ and proving its dependence on lamella thickness $\lambda_{L}$ and domain size $\lambda_{D}$.

### 4.1. Calculation Scheme

**Figure 9:**
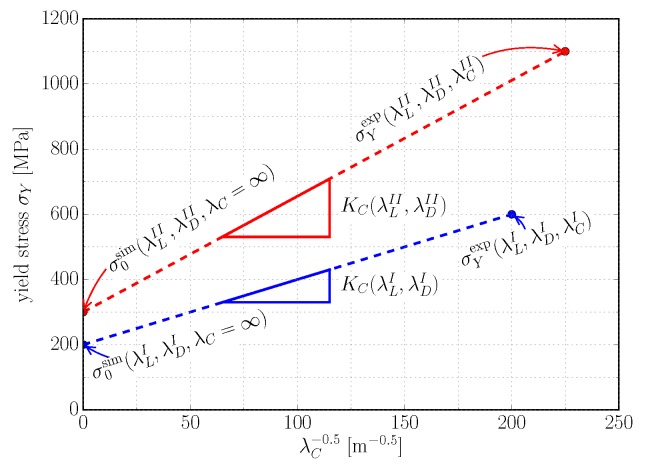
Interpolation scheme for determining $K_{C}\left(\lambda_{L}^{i},\lambda_{D}^{i}\right)$ by combination of simulation and experimental results. Applying the constitutive model of a polysynthetically twinned crystal to a polycolony RVE, yields $\sigma_{0}^{\mathrm{sim}}\left(\lambda_{L}^{i},\lambda_{D}^{i},\lambda_{C}=\infty \right)$ for a given combination of $\lambda_{L}^{i}$ and $\lambda_{D}^{i}$. With corresponding experimental results, the relation $K_{C}\left(\lambda_{L}^{i},\lambda_{D}^{i}\right)=\frac{\sigma_{Y}^{\exp}\left(\lambda_{L}^{i},\lambda_{D}^{i},\lambda_{C}^{i}\right)-\sigma_{0}^{\mathrm{sim}}\left(\lambda_{L}^{i},\lambda_{D}^{i}\right)}{\lambda_{C}^{i^{-0.5}}}$ is evaluated. Repeating this for different combinations of $\lambda_{L}$ and $\lambda_{D}$ reveals $K_{C}=f\left(\lambda_{L},\lambda_{D}\right)$.

The Hall–Petch relation in its original form $\sigma_{Y}=\sigma_{0}+KD^{-0.5}$ requires knowledge of $\sigma_{0}$ and Hall–Petch slope *K* to predict the yield stress $\sigma_{Y}$ of a polycrystalline material from its grain size *D*. In this, $\sigma_{0}$ represents the (theoretical) yield stress of a polycrystalline alloy with infinitely large grains, i.e., grain size D→∞ and thus $D^{-0.5}\to 0$. Transferring this idea to colony boundary strengthening, $\sigma_{0}$ represents the (theoretical) yield stress of a fully lamellar alloy with infinitely large colonies, i.e., without the influence of colony boundaries. Although such a microstructure can obviously not exist in reality, a model representation of it can still be created by directly applying the presented constitutive model of a polysynthetically twinned crystal—which exactly represents colonies without the influence of colony boundaries—to the polycolony RVE shown in Figure 6. Simulation results from such a model yield $\sigma_{0}^{\mathrm{sim}}$ as function of $\lambda_{L}$ and $\lambda_{D}$ and quasi represent a homogenization of a polysynthetically twinned crystals anisotropic yield stress. Being able to calculate $\sigma_{0}\left(\lambda_{L},\lambda_{D}\right)$, we may rearrange the Hall–Petch relation as follows:

(34) $$K_{C}\left(\lambda_{L},\lambda_{D}\right)=\frac{\sigma_{Y}^{\exp}\left(\lambda_{L},\lambda_{D},\lambda_{C}\right)-\sigma_{0}^{\mathrm{sim}}\left(\lambda_{L},\lambda_{D}\right)}{\lambda_{C}^{-0.5}},$$

in order to determine the Hall–Petch slope $K_{C}\left(\lambda_{L}^{i},\lambda_{D}^{i}\right)$ individually for any combination of $\lambda_{D}^{i}$ and $\lambda_{L}^{i}$ from simulated $\sigma_{0}^{\mathrm{sim}}\left(\lambda_{L}^{i},\lambda_{D}^{i}\right)$ and corresponding experimentally determined yield stress $\sigma_{Y}^{\exp}\left(\lambda_{L}^{i},\lambda_{D}^{i},\lambda_{C}^{i}\right)$. This is illustrated in Figure 9.

This interpolation scheme allows for determining $K_{C}\left(\lambda_{L}^{i},\lambda_{D}^{i}\right)$ for a certain combination of $\lambda_{D}^{i}$ and $\lambda_{L}^{i}$ by only one simulation and one experiment (although more experiments would obviously help to reveal the scatter of mechanical behavior) and if repeated for different combinations of $\lambda_{D}$ and $\lambda_{L}$ reveals the relation $K_{C}=f\left(\lambda_{L},\lambda_{D}\right)$.

### 4.2. Simulation Results

For determining $\sigma_{0}\left(\lambda_{L},\lambda_{D}\right)$ as described above, we assume $\tau_{R}$ to be 30 MPa in the γ phase. Since the domain size is reported in neither of the references gathered in Table 4 but is suspected to be correlated to the lamellar thickness [12,25], we further assume $\lambda_{D}=50\lambda_{L}$. This assumption seems reasonable evaluating lamella/domain size ratios reported for polysynthetically twinned crystals [8,19].

**Figure 10:**
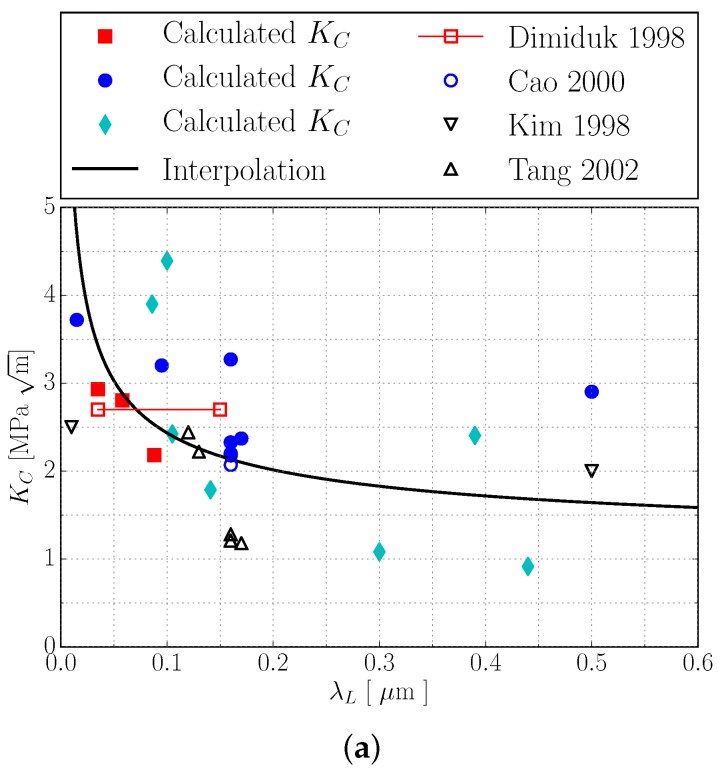

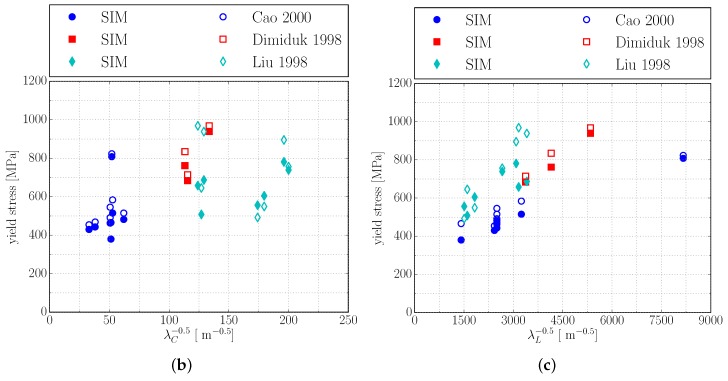
Colony boundary Hall–Petch coefficient $K_{C}$ plotted over $\lambda_{L}$. Full symbols: determined via the scheme illustrated in Figure 9; solid line: interpolation of calculated values via $K_{C}\left(\lambda_{L}\right)=K_{C,0}+K_{C,\lambda_{L}}\frac{1}{\sqrt{\lambda_{L}}}.$; open symbols: experimentally determined in [12,13,14,16]. $K_{C}$ value from [13] was determined from experiments with the indicated range of lamella thicknesses $\lambda_{L}$ (**b**,**c**): comparison of experimentally determined $\sigma_{Y}^{\exp}\left(\lambda_{L},\lambda_{D},\lambda_{C}\right)$ [10,13,14]and simulated yield stresses $\sigma_{Y}^{\exp}\left(\lambda_{L},\lambda_{D},\lambda_{C}\right)$. In simulations, colony boundary strengthening was incorporated by introducing the interpolation from Figure 10a to Equations (21) and (22). Despite the scattering of KC values, the simulated yield stresses reproduce the experimental results very well.

Figure 10a shows $K_{C}$ values determined via the above introduced scheme using the experimental results from [10,13,14] (cf. Table 4) plotted over $\lambda_{L}$.

The improvable correlation of the determined $K_{C}$ values in Figure 10a most likely results from the fact that neither domain sizes $\lambda_{D}$ nor $\alpha_{2}$ volume contents were completely reported for the underlying experiments [10,13,14]. Still, the simulations successfully demonstrate that the colony boundary strengthening coefficient $K_{C}$ must be a function of lamella thickness $\lambda_{L}$ and domain size $\lambda_{D}$ as it was suspected in [12]. In order to finally incorporate colony boundary strengthening in the model via Equations (21) and (22), a functional relation for $K_{C}$ has to be established. Since usually $\lambda_{D}\gg \lambda_{L}$ and, thus, $\frac{1}{\sqrt{\lambda_{D}}}\ll \frac{1}{\sqrt{\lambda_{L}}}$, we expect that $K_{C}$ is considerably more sensitive to changes in $\lambda_{L}$ than in $\lambda_{D}$. Thus, we assume in the following that $K_{C}$ is a function of $\lambda_{L}$ only. With this simplification, we choose the following interpolation of the calculated $K_{C}$ values in Figure 10a:

(35) $$K_{C}\left(\lambda_{L}\right)=K_{C,0}+K_{C,\lambda_{L}}\frac{1}{\sqrt{\lambda_{L}}}.$$

In this, $K_{C,0}$ corresponds to colony boundary strengthening in globular γ TiAl alloys (i.e., $\lambda_{L}=\infty$) and is, thus, assumed to be $K_{C,0}=\;$1 MPa$\sqrt{m}$ [12]. The linear coefficient $K_{C,\lambda_{L}}$ is determined to $K_{C,\lambda_{L}}=4.5\times 10^{-4}$ MPa${\left[\sqrt{m}\right]}^{2}$. The chosen functional relation $K_{C}\left(\lambda_{L}\right)$ has to be resolved to the slip and twinning systems in order to be used in Equations (21) and (22). Since no common Schmid factor can be determined for slip/twinning systems of arbitrarily oriented colonies, we use a factor of 0.3 to map $K_{C}\left(\lambda_{L}\right)$ to $k_{C}\left(\lambda_{L}\right)$. This yields:

(36) $$k_{C}\left(\lambda_{L}\right)=k_{C,0}+k_{C,\lambda_{L}}\frac{1}{\sqrt{\lambda_{L}}}=0.3\mathrm{MPa}\sqrt{m}+1.35\times 10^{-4}\mathrm{MPa}{\left[\sqrt{m}\right]}^{2}\frac{1}{\sqrt{\lambda_{L}}}.$$

Including definition (36) into Equations (21) and (22) and rerunning the simulations with the reported colony sizes $\lambda_{C}$ from Table 4 yields a good qualitative and reasonable quantitative agreement with experimentally determined yield stresses as depicted in Figure 10b,c. These results indicate that the predicted yield stress $\sigma_{Y}^{\mathrm{sim}}\left(\lambda_{L},\lambda_{D},\lambda_{C}\right)$ is not too sensitive to the deviations between calculated $K_{C}$ values and used interpolation function (35) (cf. Figure 10a), which is evident since the inverse square root $\lambda_{C}^{-0.5}$ of colony size takes comparatively small values of 25 m${}^{-0.5}$ to 200 m${}^{-0.5}$.

## 5. Conclusions

Most reported micromechanical models of polysynthetically twinned crystals were designed to capture their yield point and, thus, do not reproduce their hardening behavior for higher plastic strains. As opposed to previously reported models, the here presented hardening relations and interactions are able to reproduce all distinct features experimentally observed in [9]. This is associated with the incorporated strengthening effect through non-coplanar twins, which triggers transition between dominant deformation systems, causing changes in slope of the apparently linear hardening behavior. The presented hardening model is therefore helpful for further investigation of defect density evolution in polysynthetically twinned crystals and polycolony microstructures.

With the presented modeling approach, one can determine the Hall–Petch-coefficient $K_{C}$ to a good degree from very few experiments. Of course, the more data available, the better the computational determined value for $K_{C}$. Although the accuracy of the here determined $K_{C}$ values suffers from missing domain sizes and unknown $\alpha_{2}$ volume contents in used literature experimental data (cf. Table 4), we showed that $K_{C}$ depends on the lamellar microstructure and needs to be modeled at least as a function of the lamella thickness $\lambda_{L}$. Depending on the desired degree of detail, the domain size $\lambda_{D}$ may be added as well, but can also be disregarded as its influence is much smaller.

In summary, for the introduced approach, only a very small number of lamellar microstructures with arbitrary combinations of $\lambda_{L}$, $\lambda_{D}$ and $\lambda_{C}$ needs to be tested experimentally. This significantly simplifies the determination of the Hall–Petch coefficient $K_{C}=f\left(\lambda_{L},\lambda_{D}\right)$.

## Figures and Tables

**Table 1:** Slip and twinning systems in γ and $\alpha_{2}$ phase with morphological classification according to [6].

γ **Phase**				
**System**	**Mechanism**	**Classification**	**Index**	
$\left(111\right)\left[1\bar{1}0\right]$	ordinary slip	longitudinal	1	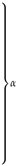
$\left(111\right)\left[01\bar{1}\right]$	super slip	longitudinal	2	
$\left(111\right)\left[10\bar{1}\right]$	super slip	longitudinal	3	
$\left(\bar{1}\bar{1}1\right)\left[1\bar{1}0\right]$	ordinary slip	mixed	4	
$\left(1\bar{1}\bar{1}\right)\left[01\bar{1}\right]$	super slip	mixed	5	
$\left(1\bar{1}1\right)\left[10\bar{1}\right]$	super slip	mixed	6	
$\left(1\bar{1}1\right)\left[110\right]$	ordinary slip	transversal	7	
$\left(\bar{1}11\right)\left[110\right]$	ordinary slip	transversal	8	
$\left(11\bar{1}\right)\left[0\bar{1}\bar{1}\right]$	super slip	transversal	9	
$\left(11\bar{1}\right)\left[\bar{1}0\bar{1}\right]$	super slip	transversal	10	
$\left(1\bar{1}1\right)\left[0\bar{1}\bar{1}\right]$	super slip	transversal	11	
$\left(1\bar{1}\bar{1}\right)\left[\bar{1}0\bar{1}\right]$	super slip	transversal	12	
$\left(111\right)\left[11\bar{2}\right]$	twinning	longitudinal	1	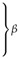
$\left(\bar{1}11\right)\left[\bar{1}1\bar{2}\right]$	twinning	transversal	2	
$\left(1\bar{1}1\right)\left[1\bar{1}\bar{2}\right]$	twinning	transversal	3	
$\left(11\bar{1}\right)\left[112\right]$	twinning	transversal	4	
$\alpha_{2}$ **Phase**				
**System**	**Mechanism**	**Classification**	**Index**	
$\langle 11\bar{2}0\rangle \left(0001\right)$	basal slip	longitudinal	1–3	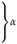
$\langle 11\bar{2}0\rangle \left\{1\bar{1}00\right\}$	prismatic slip	mixed	4–6	
$\langle \bar{1}\bar{1}26\rangle \left\{11\bar{2}1\right\}$	pyramidal slip	transversal	7–12	

**Table 2:** Material and model parameters from literature.

	Phase	Symbol	Value	Annotation	Ref.
material parameters	γ	*E*	$173.59\;\left[\mathrm{GPa}\right]-0.0342\left[T-T_{0}\right]\;\left[\frac{\mathrm{GPa}}{{}^{\circ }C}\right]$	$T_{0}=25<T<935$	[62]
		ν	$0.234+6.7\times 10^{-6}\left[T-T_{0}\right]\;\left[\frac{1}{{}^{\circ }C}\right]$	$T_{0}=25<T<847$	[62]
		$\frac{c}{a}$	$1.00356+7.2\times 10^{-6}\left[T-T_{0}\right]\;\left[\frac{1}{{}^{\circ }C}\right]$	$T_{0}=20<T<1450$	[63]
		$\gamma_{T}$	$\frac{1}{\sqrt{2}}\left[-\right]$		[5]
	$\alpha_{2}$	*E*	$147.05\;\left[\mathrm{GPa}\right]-0.0525\left[T-T_{0}\right]\;\left[\frac{\mathrm{GPa}}{{}^{\circ }C}\right]$	$T_{0}=25<T<954$	[62]
		ν	$0.295-5.9\times 10^{-5}\left[T-T_{0}\right]\;\left[\frac{1}{{}^{\circ }C}\right]$	$T_{0}=25<T<954$	[62]
		$\frac{c}{a}$	0.804≈const.	$T_{0}=20<T<1450$	[63]
	γ/$\alpha_{2}$	$\rho_{0}$	$4.219\;\left[\frac{g}{{\mathrm{cm}}^{3}}\right]-1.579\times 10^{-4}\left[T-T_{0}\right]\;\left[\frac{g}{{\mathrm{cm}}^{3}{\;}^{\circ }C}\right]$	$T_{0}=25<T<1150$	[64]
		$c_{p}$	$0.6207\;\left[\frac{J}{g{\;}^{\circ }C}\right]+1.5897\times 10^{-4}\left[T-T_{0}\right]\;\left[\frac{J}{g{{[}^{\circ }C]}^{2}}\right]$	$T_{0}=20<T<900$	[65]
		κ	$15.35\;\left[\frac{W}{m{\;}^{\circ }C}\right]+1.364\times 10^{-2}\left[T-T_{0}\right]\;\left[\frac{W}{m{{[}^{\circ }C]}^{2}}\right]$	$T_{0}=100<T<900$	[65]
		$\alpha_{t}$	$8.936\times 10^{-6}\;\left[\frac{1}{{}^{\circ }C}\right]+3.4\times 10^{-9}\left[T-T_{0}\right]\;\left[\frac{1}{{{[}^{\circ }C]}^{2}}\right]$	$T_{0}=100<T<900$	[65]
model parameters	γ	$k_{D}$	$k_{D}\left(0{\;}^{\circ }C\right)+k_{D}\left(T\right)$		[18]
		$k_{D}\left(0{\;}^{\circ }C\right)$	0.125 [MPa$\sqrt{m}$]		[18]
		$k_{D}\left(T\right)$	$\sin\left(0.00395\left[\frac{1}{{}^{\circ }C}\right]T\right)\left[2.41\times 10^{-6}\;\frac{\left[\mathrm{Pa}\sqrt{m}\right]}{{{[}^{\circ }C]}^{3.61}}\;T^{3.61}\right]$		[18]
		$k_{L}$	$k_{L}\left(0{\;}^{\circ }C\right)+k_{L}\left(T\right)$		[18]
		$k_{L}\left(0{\;}^{\circ }C\right)$	0.125 [MPa$\sqrt{m}$]		[18]
		$k_{L}\left(T\right)$	$\sin\left(0.00462\left[\frac{1}{{}^{\circ }C}\right]T\right)\left[2.64\;\frac{\left[\mathrm{Pa}\sqrt{m}\right]}{{{[}^{\circ }C]}^{1.54}}\;T^{1.54}\right]$		[18]
	γ/$\alpha_{2}$	$\nu_{0}$	$0.001\;[\frac{1}{s}]$		[18]
		*n*	50[−]		[18]

**Table 3:** Identified model parameters; ls: longitudinal slip, ms: mixed slip, ts: transversal slip, lt: longitudinal twinning, tt: transversal twinning.

	Phase	Symb	Value	Unit	System Index (cf. Table 1)	Annotation
dislocation accumulation	γ	$A_{\alpha ,0}$	$1\times 10^{9}$	[$\frac{1}{{\mathrm{mm}}^{2}}$]	α=1–3	ls
		$A_{\alpha ,0}$	$2\times 10^{9}$	[$\frac{1}{{\mathrm{mm}}^{2}}$]	α=4–6	ms
		$A_{\alpha ,0}$	$2\times 10^{9}$	[$\frac{1}{{\mathrm{mm}}^{2}}$]	α=7–12	ts
		$\rho_{\alpha ,\mathrm{sat}}^{\mathrm{dis}}$	$1\times 10^{8}$	[$\frac{1}{{\mathrm{mm}}^{2}}$]	α=1–12	all slip systems
		$p_{\alpha }$	0.05	[–]	for α=1–12	all slip systems
	$\alpha_{2}$	$A_{\alpha ,0}$	$2\times 10^{9}$	[$\frac{1}{{\mathrm{mm}}^{2}}$]	α=4–6	ms
		$A_{\alpha ,0}$	$2\times 10^{9}$	[$\frac{1}{{\mathrm{mm}}^{2}}$]	α=7–12	ts
		$\rho_{\alpha ,\mathrm{sat}}^{\mathrm{dis}}$	$1\times 10^{8}$	[$\frac{1}{{\mathrm{mm}}^{2}}$]	α=4–12	ms and ts
		$p_{\alpha }$	0.05	[–]	α=4–12	ms and ts
hardening parameters	γ	$h_{\alpha \beta }$	0	[MPa]	α=1–3 and β=1	ls by lt
		$h_{\alpha \beta }$	100	[MPa]	α=4–12 and β=1	ms and ts by lt
		$h_{\alpha \beta }$	1500	[MPa]	α=1–6 and β=2–4	ls and ms by tt
		$h_{\alpha \beta }$	300	[MPa]	α=7–12 and β=2–4	ts by tt
		$h_{\beta \beta^{{}^{\prime }}}$	0	[MPa]	β=1 and $\beta^{{}^{\prime }}=\;$1	lt by lt
		$h_{\beta \beta^{{}^{\prime }}}$	1500	[MPa]	β=1 and $\beta^{{}^{\prime }}=\;$2–4	lt by tt
		$h_{\beta \beta^{{}^{\prime }}}$	300	[MPa]	β=2–4 and $\beta^{{}^{\prime }}=\;$2–4; $\beta \neq \beta^{{}^{\prime }}$	tt by tt
		$h_{\beta \beta^{{}^{\prime }}}$	100	[MPa]	β=2–4 and $\beta^{{}^{\prime }}=\;$1	tt by lt
		$C_{\beta \alpha }$	900	[–]	β=1 and α=1–12	lt by all slip systems
		$C_{\beta \alpha }$	150	[–]	β=2–4 and α=1–12	tt by all slip systems

**Table 4:** Microstructural data from literature experiments with fully lamellar alloys. *: not reported. If no $\alpha_{2}$ was reported in corresponding reference, it was set to 10 Vol. %; the domain sizes $\lambda_{D}$ are assumed to be $50\;\lambda_{L}$

Composition	$\alpha_{2}$ Content [Vol. %]	$\lambda_{C}$ [μm]	$\lambda_{D}$ [μm]	$\lambda_{L}$ [μm]	Ref.
Ti-45.3Al-2.1Cr-2Nb	20	75	4.4 *	0.088	[13]
Ti-45.3Al-2.1Cr-2Nb	29	78	2.9 *	0.058	[13]
Ti-45.3Al-2.1Cr-2Nb	32	56	1.75 *	0.035	[13]
Ti-45.5Al-2Cr-1.5Nb-1V	10 *	260	8 *	0.16	[14]
Ti-45.5Al-2Cr-1.5Nb-1V	10 *	390	8 *	0.16	[14]
Ti-45.5Al-2Cr-1.5Nb-1V	10 *	690	8 *	0.16	[14]
Ti-45.5Al-2Cr-1.5Nb-1V	10 *	920	8.5 *	0.17	[14]
Ti-45.5Al-2Cr-1.5Nb-1V	10 *	370	0.75 *	0.015	[14]
Ti-45.5Al-2Cr-1.5Nb-1V	10 *	360	4.75 *	0.095	[14]
Ti-45.5Al-2Cr-1.5Nb-1V	10 *	380	25 *	0.5	[14]
Ti-47Al-2Cr-2Nb	10 *	65	5 *	0.1	[10]
Ti-47Al-2Cr-2Nb	10 *	62	19.5 *	0.39	[10]
Ti-47Al-2Cr-2Nb-0.15B	10 *	33	22 *	0.44	[10]
Ti-47Al-2Cr-1.8Nb-0.2W-0.15B	10 *	31	15 *	0.3	[10]
Ti-47Al-2Cr-1.8Nb-0.2W-0.15B	10 *	25	7.05 *	0.141	[10]
Ti-46Al-2Cr-1.8Nb-0.2W-0.15B	10 *	26	5.25 *	0.105	[10]
Ti-47Al-2Cr-1Nb-1Ta	10 *	60	4.3 *	0.086	[10]
